# Laparoscopic Lateral Suspension (LLS) for the Treatment of Apical Prolapse: A New Gold Standard?

**DOI:** 10.3389/fsurg.2022.898392

**Published:** 2022-05-12

**Authors:** Patrick Dällenbach

**Affiliations:** Department of Pediatrics Gynecology and Obstetrics, Division of Gynecology, Urogynecology unit, Geneva University Hospitals, Geneva, Switzerland

**Keywords:** pelvic organ prolapse, apical prolapse, sacrocolpopexy, lateral suspension, hysteropexy

## Abstract

Nowadays, the gold standard to treat apical pelvic organ prolapse (POP) is laparoscopic sacrocolpopexy (LSCP). However, LSCP is a difficult procedure associated with rare but potentially severe complications. Promontory dissection may expose to potential life-threatening intraoperative vascular injuries, and sacral roots or hypogastric nerve damage. There are also a few case reports of spondylodiscitis with consecutive lumbar vertebra bone erosion. Laparoscopic lateral suspension (LLS) with mesh is an alternative technique for apical POP repair. It lowers perioperative risks by avoiding sacral promontory preparation. Recent studies show similar anatomical and functional outcomes to LSCP, with the advantage of better preserving the vaginal axis. Moreover, LLS is well suited for hysteropexy which is important as an increasing number of women prefer uterine preservation during POP surgery. In this article, we discuss both techniques, and we share our opinion on a novel perspective in the treatment of apical POP with uterine preservation.

## Introduction

Pelvic organ prolapse (POP) is a condition affecting up to 40% of an outpatient setting and causes impaired body image and decreased quality of life (1–3)*.* Most women suffer in silence and shame (4, 5). By the age of 85, 19% of women will have undergone a surgical cure for their prolapse (6). POP are hernias of the pelvic floor involving three compartments: anterior-related to the bladder, apical-related to the uterus, and posterior-related to the rectum. The gold standard to treat apical prolapse is sacrocolpopexy (SCP) (7). However, SCP is a difficult procedure with potentially severe complications. Promontory dissection exposes to potential life-threatening vascular injuries, spondylodiscitis, hypogastric nerve impairment (resulting in bladder or bowel dysfunction), or sacral roots damage (8–11). This step of the procedure may be challenging, especially in obese patients. Therefore, there is interest for alternative pelvic floor repair procedures which would guarantee the same anatomical and functional outcomes with less perioperative risks. Laparoscopic lateral suspension (LLS) with mesh is an alternative approach for apical POP repair avoiding promontory dissection, thereby lowering perioperative risks. Recent series show comparable results (Table 1). Moreover, LLS is well suited for hysteropexy which is an important feature as many women prefer uterine preservation during POP surgery (12, 13). The objective of this article is to show the advantages of LLS over LSCP and set a new gold standard for apical POP with uterine preservation.

**Table 1:** LLS and RALLS main studies.

Study	N	Mean follow-up (months)	Objective^a^ success rate(%)	Subjective^b^ success rate (%)	Reoperation for recurrence (%)	Laparotomy conversion (%)	Perioperative complications (%)	Mesh erosion (%)
LLS Dubuisson 2011	218	17.8	86.2	NA	4.6	0	1.8^c^	6
LLS Martinello 2019	48	24	>80	NA	6.3	0	0	0
Chatzioannidou 2021 standardized LLS	88	40	87.3	96.2	5.1	0	0	0
**Simoncini et al. 2016**- RALLS (82)	40	1	100	NA	NA	0	0	2.5
LLS (60) and RALLS (60) Mereu et al 2019	120	24	94.2	89	6.4	0	0	0.8
RALLS Dällenbach et al 2021	54	33	83.3	77.2	9.3	0	0	0

^a^ Objective success defined by the anatomical correction of the prolapse during the clinical examination.

^b^ Subjective success defined as the patient satisfaction measured by PGI-I (Patient Global Impression of Improvement for urogenital prolapse).

^c^ 1 bladder perforation, 1 abdominal wall hematoma, 1 bowel obstruction due to trocar hernia, one umbilical trocar hernia.

## Background and History

### Sacrocolpopexy (SCP)

Historically, SCP was developed to treat recurrent vaginal vault prolapse. It was performed abdominally at the end of the 1950s, adapted to laparoscopy in the 1990s, and performed with robotic assistance since 2004 (14–17)*.* It is nowadays the gold standard to treat apical POP (7). In case of apical POP in women without previous hysterectomy, surgeons often performed the procedure with associated hysterectomy. Studies showed that the rate of mesh exposure was nearly six times lower when the uterus was preserved, and progressively, total hysterectomy was replaced by supracervical hysterectomy to reduce this risk (18). To meet the expectations of women who increasingly prefer to keep their uterus, a symbol of femininity (12, 13), the technique started to be performed with uterine preservation (19).

### Evolution of the Technique

Sacral colpopexy was initially developed by interposing a prosthesis between the apex of the vagina and the sacrum. In order to maintain a physiological orientation of the vagina, some authors have fixed the prosthesis directly at level S3-S4 (20). After experiencing threatening haemorrhages, the prostheses were fixed a little higher on the promontory, where the middle sacral artery was well identified and damage avoided (8)*.* In order to limit the risk of the prosthesis detaching from the vagina, it was gradually placed along the entire length of the rectovaginal septum and the vesico-vaginal septum to limit the risk of subsequent cystocele (9). Author agrees that today, in the event of significant prolapse of anterior and posterior compartment, a mesh should be placed deep in the vesico-vaginal and in the recto-vaginal septum until the levator ani muscles (21). However, there remains a certain heterogeneity of practices in various centres.

### Outcomes and Safety of SCP

Results of SCP, whether abdominal, laparoscopic, or robotic, all show very high cure rate during short term follow-up with good results at a longer term. In a large review dated 2004 including abdominal (ASCP) and laparoscopic sacrocolpopexy (LSCP), Ingrid Nygaard described success rates between 78 and 100 percent for follow-ups from six months to three years (9). In a more recent review of LSCP and robotic sacrocolpoepxy (RSCP), Richard Lee described success rates of around 90% for both techniques with an average follow-up of 26 months (22). In a review analysing the longer-term results of ASCP, Ingrid Nygaard described reduced success rates of around 70%–75% with a 7-year follow-up (23). Sarlos and al in a LSCP study including 101 patients described an objective success rate at one year of 98% which decreased to 83.8% at five years (24, 25). The 2016 Cochrane review describes better results for SCP compared to the vaginal route in the treatment of apical prolapse. However, the differences in absolute values are relatively modest, since success rates for SCP are estimated at 93% in the short term, versus 86% for vaginal procedures. Reoperation for recurrence was of 4% in SCP and only between 5 and 18 percent for vaginal procedures (7).

Although LSCP has been performed for nearly 30 years and is considered the gold standard for apical prolapse, it remains less widely used than the vaginal route (26). The main reason for disaffection for this technique is the perceived difficulty of this procedure compared to the ease of vaginal techniques. Claerhout in Belgium evaluated the learning curve of LSCP and found that it takes around 60 procedures to ensure anatomical success and limit the risk of complications. Operative time decreased rapidly after the first 30 procedures and reached a steady state after 90 procedures (27). Alex Mowat described in an article that a structured program could reduce this learning curve (28)*.* The advent of robotic surgery facilitated the realization of laparoscopic sutures, and could help reduce this learning curve. However, studies on this subject report relatively similar numbers of 50 to 75 operations required before mastering the technique (29, 30).

Complications of SCP are rare but potentially severe. Ingrid Nygaard’s comprehensive review described not only bladder (3.1%) and rectal wounds (1.6%) but above all haemorrhages or transfusions in 4.4% of cases. Mesh erosion rate was 3.4%. This review included many open ASCP which could explain the increase in bleeding complications (9). More recent studies using the minimally invasive route described, in addition to bladder and rectal wounds and haemorrhagic complications, the conversion to laparotomy in nearly 4% of cases (24, 31). Reoperation rate for mesh erosion were also close to 3% in these studies. In one of the largest series of LSCP published today comprising 1,238 procedures, the Clermont-Ferrand team described 2.7% of severe complications including hematomas, peritonitis and complications related to prostheses (32).

In Richard Lee’s review, there was also a case of spondylodiscitis, which is a complication occasionally described after SCP and which can be destructive to the surrounding bone (22). There are several case reports of this complication in the medical literature (33, 34). In the same review by Lee, the rate of de novo stress urinary incontinence was between 0 and 30% after SCP, averaging around 9% (22).

### Laparoscopic Lateral Suspension (LLS)

LLS is not a new technique. It was developed by laparotomy in the 1960s by Kapandji (35), and like SCP was adapted to laparoscopy by Cornier in 1994 (36), then developed by Dubuisson in the 2000s (37–41). The technique was first performed with robotic assistance in 2014 by ourselves, with improvements authorized by robotic ergonomics allowing less scars compared to the laparoscopic technique (42). Since the first description of the technique, the difference with SCP lies in the fact that LLS was a prosthetic suspension of the uterine isthmus (hysteropexy) in contrast to the suspension of the vaginal vault for SCP. However, in the first Dubuisson series, subtotal hysterectomy was also frequently performed. In a French randomized trial on 50 patients published in 1983 by comparing ASCP with lateral suspension according to Kapandji’s technique, the results obtained for lateral suspension were better in terms of prolapse and urinary incontinence (43). Paradoxically, SCP has been the preferred and most widespread technique over the past 40 years and is accepted as the gold standard today.

### Evolution of the Technique

Kapandji’s initial description was a colpo-isthmo-cystopexy using a transverse band that he attached to the aponeurosis of the oblique muscle opposite the anterior and superior iliac spine. The strip used was 2 cm wide Crinoruban® (polyamide) or Teflon® (Polytetrafluoroethylene), or sometimes skin, and was attached to the posterior wall of the bladder, to the vaginal fascia, and to the uterine isthmus by Tergal® (polyester) threads. The fixation of the aponeurosis of the oblique muscle was done by a point of Catgut n°1. The technique associated a section and tensioning of the round ligaments plicated forwards, and a Douglassoraphy with plication of the uterosacral ligaments (35).

In 1994, Cornier and Madelanat described for the first time a laparoscopic hysteropexy according to Kapandji in 7 patients (36). The technique was similar, but with fixation of a Mersilene® (polyester) prosthesis comprising of a 2 × 2 cm anterior tongue, and 3 cm wide and 10 cm long lateral arms, fixed to the iliac spines. Unlike Kapandji’s original technique, they simply fixed the strip to the vagina and the uterine isthmus, without fixing it to the posterior wall of the bladder. The anterior tongue was secured by transfixing vaginal stitches tied in the vagina with nylon 00. It was also associated with a Douglassoraphy and plication of the uterosacral ligaments by a vaginal transfixing thread of nylon 00. The prosthesis was then fixed by a nylon thread to the aponeurosis of the oblique muscle at the level of the iliac spines.

**Figure 1:**
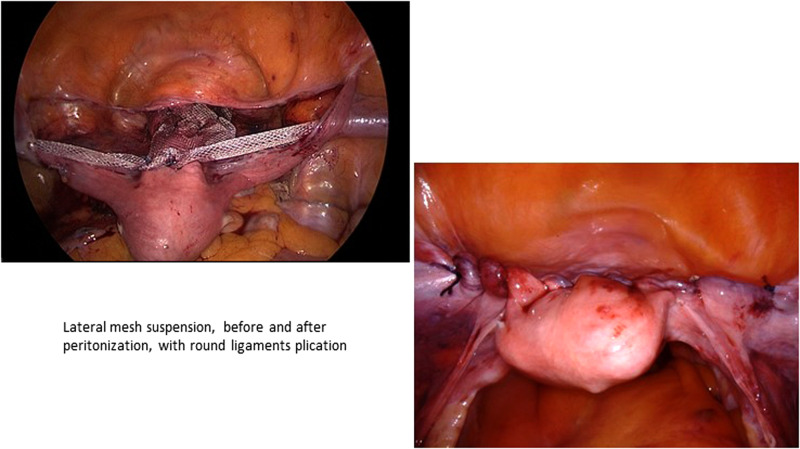
Lateral mesh suspension, before and after peritonization, with round ligaments plication.

The technique was then further developed and described by Dubuisson by modifying the shape of the prostheses with an anterior tab 6 cm long and 4 cm wide, attached to the vesico-vaginal fascia, with fixation entirely by laparoscopy. He initially used two lateral suspension prostheses, one anterior and one posterior, with two pairs of lateral arms fixed a little higher, about 5 cm above the anterior superior iliac spine, giving an even more physiological vaginal angulation (37, 38)*.* He then developed the technique by placing a single anterior prosthesis with a 6 × 4 tab in the vesico-uterine space, associated either with a posterior prolapse cure by the vaginal route (posterior colporraphy), or with the placement of a posterior prosthetic tension free patch (39–41). He initially fixed the lateral arms to the abdominal fascia and progressively abandoned it creating a tension free sub peritoneal passage. He fixed the lateral arms to the peritoneum with absorbable tackers (AbsorbaTack™fixation device by Medtronic, Minneapolis,MN, USA). He subsequently used a prosthesis of polyester (Mersilene® by Ethicon), then of polypropylene (Gynecare Gynemesh®) and finally developed a prosthesis of macroporous polypropyene covered with titanium (TiLOOP® “Prof Dubuisson”® 9 × 41.5 cm, 65 g/m², pfm medical, Germany). The prosthesis was fixed to the vesico-vaginal fascia and to the isthmus uteri with non-absorbable threads of Ethibon ® (polyester sutures) and sometimes with synthetic glues (Glubran®). We have further standardized the Dubuisson technique and published two series of laparoscopic and robotic techniques using only absorbable sutures to fix the Ti-LOOP prosthesis to the vaginal fascia (Figure 1). In case of associated rectocele, the treatment of the posterior compartment was done vaginally only when required (44, 45). These further developments reduced the vaginal mesh erosion rate to zero without impairing effectiveness. We believe it is unnecessary to use non absorbable polyester sutures to fix the mesh to the vesico-vaginal fascia as the subsequent fibrosis fixes the mesh. Non absorbable sutures may sometimes transfix the vaginal wall and carry bacteria to the mesh material enhancing the risk of erosion.

### Outcomes and Safety of LLS

Results of the main studies are summarized in Table 1. They are very similar to the ones of SCP, with around 90% of success at short term follow-up and over 80% of success at 3 years. Thus far longer follow-up studies are unavailable to compare with SCP. An important feature in all the LLS series is the absence of major perioperative complications, in particular no severe bleeding and no conversion to laparotomy. In our experience, the learning curve is also shorter compared to SCP, but we have no comparative study to prove it. We believe 10–15 cases to be enough for mastering the technique for a trained surgeon in laparoscopic suturing. Some protagonists of SCP criticize the prosthetic lateral suspension for not treating the posterior compartment well. However, the global rate of recurrence is very low and comparable to SCP. We agree that in women for whom the posterior compartment was not treated initially, some developed posterior rectocele. However, this number remains low, and few women (between 2 and 7%) required a second surgery for this problem (39, 44–46). The mesh erosion rates were around 5% for Dubuisson’s initial series, and between 0 and 2.5% in recent reports, which is comparable to the ones described for SCP (Table 1). As previously stated, it was zero in studies using Ti-LOOP® macroporous titanised mesh with only absorbable sutures to fix the mesh on the vesico-vaginal fascia. We have analysed risk factors for mesh erosion in a previous study, and showed the importance of customizing use of mesh material (47). As discussed, avoiding non absorbable suture to fix the mesh to the vesico-vaginal fascia may also decrease the risk of mesh erosion by limiting access of bacteria to the prosthesis.

## POP Distribution

Prolapse surgery should ideally correct all the pelvic floor defects. Many surgeons have aimed to do this, but in reality pelvic floor alterations are so complex that most of our surgical techniques are defect compensation approaches rather than actual repair**.** Knowing the distribution of defects can allow us to better adapt our repair techniques. We have shown in a series of 326 patients representing of a cohort of 1,811 women consulting for surgical correction of genital prolapse over a period of 20 years, that the anterior compartment is most frequently affected and often in association with the middle compartment (48). In this study we also showed that a previous hysterectomy increased the risk of developing posterior compartment prolapse. This is probably due to the section of the uterosacral ligaments (De Lancey level 1) which participates in the support of the posterior compartment (49). Other authors found similar distributions between the compartments, with a higher proportion of prolapse of the posterior compartment (50)*.* If we consider their populations in detail, we realize that a significant proportion of women had undergone prior hysterectomy, which is consistent with our observations. If the defects of the anterior and apical compartment are most often associated, prosthetic lateral suspension is the treatment of choice as it concomitantly compensates for these two defects.

## Hysteropreservation During POP Surgery

Historically, POP surgical procedure with native tissue repair included a vaginal hysterectomy. As we have seen before, SCP was initially validated for vaginal vault prolapse. Most SCP procedures nowadays still include a hysterectomy, and supracervical hysterectomy is preferred to limit the risk of erosion. However, an increasing number of women prefer uterine preservation as it represents a symbol of femininity (12, 13). Moreover, hysterectomy is probably an unnecessary act during POP surgery, and may increase the risk of posterior compartment prolapse (48). Recent studies also show a benefit of uterine preservation by reducing operating time and blood loss without affecting outcome (18, 51). Another advantage of hysteropreservation is to avoid uterine morcellation which lengthens the operating time, increases the risks, and can in some cases disseminate abnormal uterine tissue. Therefore, we believe hysterectomy should only be performed in case of genital prolapse with an underlying uterine pathology. LLS was developed for hysteropexy and is well suited for uterine conservation. The lateral suspension follows the natural ligament suspension of the uterus thus providing a more physiological orientation of the vaginal axis (52) than during SCP, which deflects it to the right and a little backwards.

## Posterior Mesh and Risk of Erosion

The Cochrane review shows that the posterior compartment is best treated vaginally without a prosthesis with a simple posterior colporraphy (53). We showed previously that introduction of prosthetic material in the rectovaginal septum causes a fivefold risk of prosthetic erosion (47). This observation was corroborate by other authors (38, 54)*.* We hypothesized that it might be due to a different vascular supply of the posterior vaginal wall, or due to dissection close to the vaginal wall thereby avoiding rectal injury. These elements have to be taken into account when performing a pelvic floor repair and choosing SCP, during which an anterior and posterior prosthesis is generally placed. From our point of view, it would suffice to place an anterior mesh and treat the posterior compartment vaginally, which is the case with LLS in most centres nowadays. As discussed, genital prolapses most often affect the anterior and middle compartment, so it does not seem necessary to concomitantly treat the posterior compartment. Some authors will argue that a preventive correction is useful to limit the risk of recurrence. This must be weighed against the risk of complications related to prostheses in the posterior compartment. If the rectocele is not clinically significant, we choose a shared decision approach to correct it later only if it becomes symptomatic. Indeed, cures for rectoceles can sometimes be accompanied by dyspareunia (55). Since prolapse surgery is above all a functional surgery, the goal is to relieve the symptoms without necessarily seeking for perfect anatomical correction. In our experience, the need to correct a rectocele at a later stage remains infrequent and if required, the cure provided by the vaginal route is simple and rapid.

## Pelvic Organ Prolapse (POP) and Stress Urinary Incontinence (SUI)

A similar reasoning applies with regard to urinary incontinence, where we only simultaneously correct if clinically relevant. In the event of occult urodynamic SUI, or mild SUI, we discuss a two-stage intervention with the patient if necessary. In our experience, with good pre-operative explanations, this strategy is well understood and approved. We believe this saves patients from unnecessary gests and their possible complications. An increasing number of urogynecologists are adopting this strategy with regard to occult urinary stress incontinence, whereby they treat as a second step after repairing the prolapse only if it becomes symptomatic (56, 57). It is sustained by our recent studies. In our RALLS study, 60% of women with preoperative SUI were cured after the operation and there were only 5.9% of de novo SUI. In our LLS series, 40% were cured with the POP surgery alone (44). In other LLS series, de novo SUI was only 2.5% (46) and 3.7% (39), which is also less than the average 9% described for SCP (22).

## Robotic Assistance

Both techniques (SCP and LLS) have been reproduced by robot-assisted laparoscopy. Robotic assistance offers many advantages, in particular 3D vision which allows for clean bloodless dissections. Several reviews show similar results of RSCP in relation to laparoscopy or the abdominal route (58). For lateral suspension, there is still little literature, but it is also very promising. The robot makes it possible to correct ergonomic problems and has enabled us to reduce the number of scars. It allowed us to place the working trocars very laterally, enabling removal of the prosthetic braces by the same route, rather than by additional supra-iliac incision, such as in the standard laparoscopic technique described by Dubuisson. The preliminary results do not show any difference with the laparoscopic approach encouraging the use of the robot in this restorative surgery (45, 46, 59).

## Discussion

Although we contrast SCP and LLS in this article, we believe these two POP repair techniques are complementary. Based on our expertise, prosthetic lateral suspension adapts particularly well to hysteropexy, and SCP remains a better option for vaginal vault prolapses. It is important that pelvic floor surgeons master both techniques in order to be able to manage any situation for best patient outcome. In this way, they may better adapt and reduce the perioperative risks when faced with an intraoperative difficulty. For example, dissection of the promontory can be challenging in obese patients, or in the case of vascular anatomical variations, and lateral suspension may represent a safer alternative. On the other hand, a high adhesion status in the right iliac fossa after appendicitis may require dangerous adhesiolysis to access the lateral strip, and SCP may in this case provide lower risks.

However, we attempt to demonstrate in this article that prosthetic lateral suspension is safer and easier to perform, with some advantages over SCP which can be summarized as follows.

Firstly, it avoids the risks associated with the dissection of the promontory, especially the risk of haemorrhage and of spondylodiscitis which can threaten patient prognosis. Contrary to SCP series, which report a 4% risk of laparotomy, there was no such occurrence available in all of the LLS series. Moreover, the learning curve for this technique also seems faster to us.

LLS is also particularly suitable for uterine preservation, which is chosen by many women today. Hysteropexy by prosthetic lateral suspension follows the anatomical attachments of the uterus and makes it possible to preserve the vaginal axis (52). This can help maintain a normal suburethral support and potentially prevent the risk of de novo urinary incontinence, which seems less with LLS than with SCP. This remains however to be demonstrated by comparative studies.

As the distribution of genital prolapse mainly affects the anterior and middle compartments, LLS is perfectly suited to this anatomical situation. The absence of a mesh in the posterior compartment may reduce the risk of subsequent mesh erosion, thereby avoiding a number of unnecessary operations and their possible complications. Since the posterior compartment is best treated vaginally, it can be treated by standard vaginal surgery during the same operation if there is a significant rectocele, or later, if it appears secondarily at postoperative follow-up. Avoiding prophylactic posterior colporraphy may reduce the risk of dyspareunia.

In conclusion, for all these robust reasons stated above, we believe LLS should be upgraded and considered as the new gold standard for treating apical POP with a healthy uterus.

## Data Availability

The original contributions presented in the study are included in the article/**Supplementary Material**, further inquiries can be directed to the corresponding author/s.
